# The Role of ECG-Gated CT in Patients with Bicuspid Aortic Valve Replacement: New Perspectives in Short- and Long-Term Followup

**DOI:** 10.5402/2013/826073

**Published:** 2012-10-23

**Authors:** Massimiliano Sperandio, Chiara Arganini, Alessio Bindi, Armando Fusco, Carlo Olevano, Fabio Bertoldo, Andrea Romagnoli, Luigi Chiariello, Giovanni Simonetti

**Affiliations:** ^1^Dipartimento di Diagnostica per Immagini, Imaging Molecolare, Radiologia Interventistica e Radioterapia, Fondazione Ospedaliera Policlinico “Tor Vergata”, Viale Oxford 81, 00133 Roma, Italy; ^2^Dipartimento di Cardiochirurgia, Fondazione Ospedaliera Policlinico “Tor Vergata”, Viale Oxford 81, 00133 Roma, Italy

## Abstract

The aim of our study was to compare the results of the TTE (transthoracic echocardiography) with the results obtained by the ECG-gated 64 slices CT during the followup of patients with bicuspid aortic valve (BAV), after aortic valve replacement; in particular we evaluated the aortic root and the ascending aorta looking for a new algorithm in the followup of these patients. From January 1999 to December 2009 our attention was focused on 67 patients with isolated surgical substitution of aortic valve; after dismissal they were strictly observed. During the period between May and September 2010, these patients underwent their last evaluation, and clinical exams, ECG, TTE, and an ECG-gated-MDCT were performed. At followup TTE results showed an aortic root of 36.7 ± 4 mm and an ascending aorta of 39.6 ± 4.8 mm. ECG- gated CT showed an aortic root of 37.9 ± 5.5 mm and an ascending aorta of 43.1 ± 5.2. The comparison between preoperative and postoperative TTE shows a significant long-term dilatation of the ascending aorta while the aortic root diameter seems to be stable. ECG-gated CT confirms the stability of the aortic root diameter (38.2 ± 5.3 mm versus 37.9 ± 5.5  mm; <0.0001) and the increasing diameter value of the ascending aorta (40.2 ± 3.9 mm versus 43.1 ± 5.2 mm; *P* = 0.0156). Due to the different findings between CT and TTE studies, ECG-gated CT should no longer be considered as a complementary exam in the followup of patients with BAV, but as a fundamental role since it is a real necessity.

## 1. Introduction

Bicuspid aortic valve is an autosomal dominant hereditary disease with incomplete penetrance and represents the most common congenital disease that can be found in the general population, with prevalence between 1% and 2%. However, a threefold higher male predominance one was observed, suggesting that this disease may be related to an X-linked hereditary [1].

This structural alteration seems to be a consequence of an abnormal developmental process of the aortic cusps during embryogenesis [2–9].

Patients with bicuspid aortic valve have a higher morbidity and mortality owing to the associated cardiovascular complications. 

Aortic coarctation, dilatation, and consequent aneurysm or dissections of the ascending aorta are very common in this group of patients.

Aortic stenosis/insufficient valve is very common in these patients and it is related to gradual calcification of the valve. In these patients a valve replacement is necessary and the surgical rational is similar to the valve replacement of the tricuspid one. It is generally accepted that replacement of the ascending aorta should be performed, at the time of aortic valve replacement, when the aortic diameter exceeds 40–45 mm; however, it is still open to doubt which procedure should be performed and if it should be performed, especially in young patients [10]. In fact a dilatation of the ascending aorta could occur, even after the valve replacement, so the followup of the aorta in these patients is needed. 

Echocardiography is considered the first standard examination for the diagnosis and the followup of this disease, especially if performed via transthoracic approach, with sensibility of 87% and specificity of 91%.

ECG-gated CT has been shown to provide accurate information about the aortic valve and root and may be considered as an alternative imaging technique [11–13].

The aim of our study is to compare the results of the TEE (transthoracic echocardiography) with the results obtained by the ECG-gated 64 slices CT during the followup of patients with BAV, after aortic valve replacement; in particular we evaluated the aortic root and the ascending aorta looking for a new algorithm in the followup of these patients. 

## 2. Materials and Methods

### 2.1. Population

From January 1999 to December 2009, 369 patients with bicuspid aortic valve were submitted to surgical treatment by Cardiac Surgery Division of Tor Vergata University Foundation.

In particular our attention was focused on 67 patients with isolated surgical substitution of aortic valve (48 male, 19 female; mild age of 61 ± 15, min age 21, max age 88), selected for clinical and instrumental preoperative and postoperative studies with echo-cardiac and MDCT exams, on the bases of ascending aortic diameters <45 mm at the moment of surgical treatment, absence of genetic disease (e.g., Marfan syndrome), general CT exam contraindications, and also for geographical territory distribution. 

Preoperatory characteristics of this selected population of patients are shown in Table 1.

The study was approved by the local ethics committee and all the patients provided informed consent to the examination.

An echo-cardiac transthoracic exam (Table 2) and MDCT (Table 3) were performed before surgical treatment.

A preoperative coronary study was also performed by means of a conventional coronarographic study in 60 patients and a MDCT in 7 patients.

All these patients underwent surgical implanted valves; in particular, 30 of them received a biological prosthesis (Lifesciences Edwards C-E Perimount) and 37 of them a mechanical prosthesis (24 Sorin Bicarbon, 12 CarboMedics, 1 St.Jude Medical).

After dismissal, all of these patients were strictly observed with clinical exams, ECG, transthoracic echocardiography, and in selected cases with MDCT. 

### 2.2. Followup

During the period between May to September 2010, these patients underwent their last evaluation, and clinical exams, ECG, transthoracic echocardiography and an ECG-gated-MDCT were performed.

### 2.3. Echocardiography

An echo-cardiac transthoracic exam was performed in order to measure the diameter of the aortic root, sinotubular junction, and ascending aorta.

A measurement of parietal thickness, telediastolic and telesystolic left ventricular diameter and left ventricular ejection fraction was also obtained.

As regards the prosthesis aortic valve, we evaluated transvalvular maximum and medium gradients and the presence of reflux; we also studied the other valves and systolic pulmonary arterial pressure.

### 2.4. CT

MDCT was also performed before surgical treatment to measure the aortic root diameter, sinotubular junction, and ascending aorta (at the bifurcation of pulmonary trunk) and proximal aortic arch. An evaluation of the aortic valve was also made.

Cardiac frequency <75 bpm was required for the execution of the examination; in the case of higher frequency a beta blocker was administrated (5 mg propranolol ev.).

### 2.5. Acquisition Protocol

CT exams were performed with a 64-slice CT scanner (LightSpeed VCT, General Electric Medical System, Milwaukee, WI, USA), and by a retrospective synchronization technique.

A preliminary unenhanced scan was done in order to determine the scan extent and calculate the calcium score (SmartScore protocol). The acquisition stack extended from the pulmonary apex to the diaphragm inferiorly. A second image stack was then acquired after intravenous administration of iodinated contrast material using a dual-head automated injector (Stellant, MEDRAD, Pittsburgh, PA, USA). A dose of 90 mL of nonionic iodinated contrast material (Iomeron 400, Bracco, Milan, Italy) was administered through an 18-gauge needle cannula placed in an antecubital vein, followed by a 40 mL saline solution injection, both at a rate of 5 mL/s. To synchronize the acquisition start with the arrival of the contrast agent in the aortic root, the bolus-tracking technique was used. Parameters for the contrast-enhanced scan were beam collimation 64 × 0.625 mm, slice thickness 0.625 mm, reconstruction increment 0.625 mm, table feed 2.9 mm/rotation, tube rotation 0.35 s, tube voltage 120 kV, dose modulation protocol (intensity 140–750 mA), and craniocaudal scan direction. Scan duration was 10–12 s. Image reconstruction was carried out using 75% of the cardiac cycle, corresponding to the R-R interval or end diastole.

Adsorbed dose was 8–10 mSv.

### 2.6. Image Analysis

The data-set was sent to a workstation console and elaborated using a dedicated software (Advantage Windows with CardioIQ Software, version 4.4; GE healthcare, Milwaukee, USA) to get Multiplanar reconstruction (MPR), Maximum intensity projection (MIP), and volume rendering (VR) images.

### 2.7. Statistical Analysis

All the data was entered into a database for statistical processing. The data was expressed as means plus one standard deviation (SD) or as percentages. The comparison between groups was obtained using the *χ* ^2^ test and the Student's *t*-test, as appropriate. Statistical significant was set at *P* < 0.05.

## 3. Results

During the period between May to September 2010, these patients underwent their last evaluation; clinical exams, ECG, transthoracic echocardiography, and an ECG-gated-MDCT were performed.

The average time of the followup was 55 ± 35 months (range 9–137 months, median 44 months).

Table 4 shows all the clinical characteristics of the population during the followup.

TTE results show an aortic root of 36.7 ± 4 mm and an ascending aorta of 39.6 ± 4.8 mm (Table 5).

ECG-gated CT shows an aortic root of 37.9 ± 5.5 mm and an ascending aorta of 43.1 ± 5.2 (Table 6).

The comparison between preoperative and postoperative EET shows a significant long-term dilatation of the ascending aorta (37.5 ± 4.4 mm versus 39.6 ± 4.8 mm; *P* < 0.0001), while the aortic root diameter seems to be stable (36.4 ± 4.1 versus 36.7 ± 4.1 mm; *P* < 0.0001).

All the patients underwent ECG-gated CT and none of them were excluded during the examination. 

**Figure 1:**
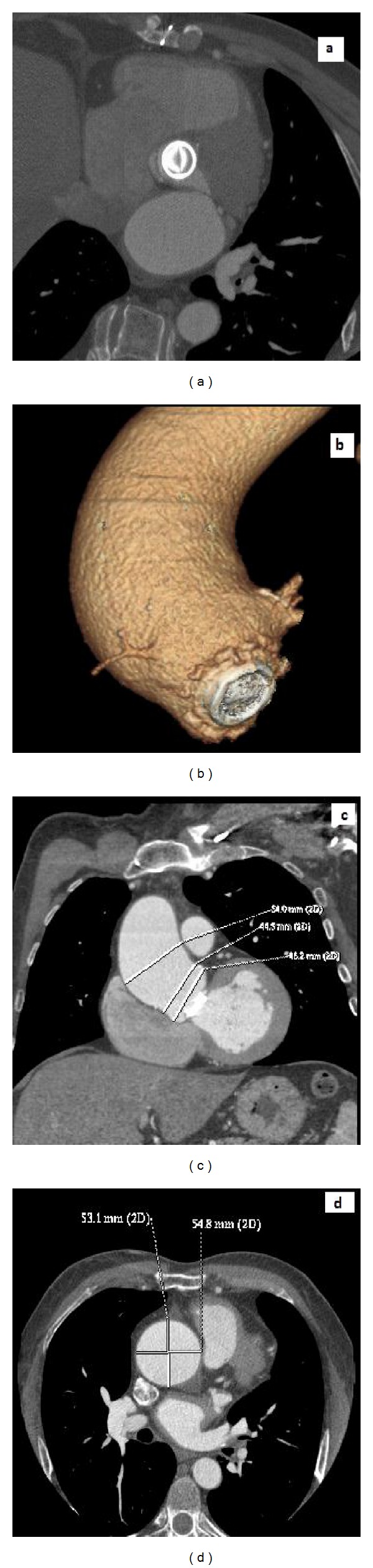
A 50-year male patient with mechanical aortic valve replaced in 1999; (a)-(b) MPR axial reconstruction and volume rendering reconstruction show prosthesic valve; (c)-(d) MPR reconstruction document dilatation of aortic root, sino-tubular junction and ascending aorta.

ECG-gated CT confirms the stability of the aortic root diameter (38.2 ± 5.3 mm versus 37.9 ± 5.5 mm; <0.0001) and the increasing diameter value of the ascending aorta (40.2 ± 3.9 mm versus 43.1 ± 5.2 mm; *P* = 0.0156). In particular, we noticed that in 7 (10.4%) patients, the diameter of the ascending aorta was ≥50 mm (Figure 1). Comparing this group of patients with the rest of the population we discovered that the only statistical difference was in the period of followup; in fact these 7 patients have been observed for a period longer than 60 months.

ECG-gated CT acquisition allowed us an evaluation of the coronary tree in all patients: 43 (64.1%) patients did not show coronary stenosis, while 21 (31.3%) of them had a nonsignificant coronary stenosis (<50%) and 3 (4.4%) had a significant coronary stenosis (≥50%).

## 4. Discussion

Bicuspid aortic valve (BAV) is the most common congenital cardiac malformation, affecting 1-2% of the population, with strong male predominance [14]. It has been recognized as a syndrome incorporating aortic valve disorders and aortic wall abnormalities. These intrinsic defects in the aortic wall end up in late complications such as aortic root dilatation, ascending aortic aneurysm, often asymmetric [15], and dissection. 

 Because these complications, with a potentially fatal outcome, will develop in approximately one third of all patients with a bicuspid aortic valve, it is of the utmost importance to correctly detect and treat this type of congenital malformation, and also to follow these possible complications over time [16]. For this reason, different surgical options have been developed for these patients, including not only aortic valve replacement but, in some cases, also concomitant prophylactic surgery of the aorta. According to the guidelines of the American College of Cardiology and of the American Heart Association, patients with BAV should undergo elective repair of the aortic root or replacement of the ascending aorta, at the time of aortic valve replacement, if the diameter of these structures exceeds 45 mm [17]. In fact, the risk of combined surgery on the aortic valve and ascending aorta is almost comparable to the risk of isolated valve replacement, but it is considerably lower than the risk of a possible resurgery in election, or even worse, in emergency. 

In case of only aortic valve replacement, a close followup is required after surgery, to evaluate aortic dilatation and to avoid late complications. Indeed, in patients with BAV without dilatation of the ascending aorta, even successful valve replacement is not able to prevent long-term dilatation of the ascending aorta. Transthoracic echocardiography (TTE) is the first standard examination during the followup of these patients [18]. Currently, in patients with previous BAV and aortic valve replacement, transthoracic echocardiographic followup is performed twice a year and it is integrated with angio-CT in the case of initial signs of aortic disease or of suspicious echocardiographic findings. Followup evaluation includes the state of the heart chambers, the functioning of prosthetic aortic valve, and the size of thoracic aorta. TEE is an excellent method for studying the aortic root, although this technique has some limitations: it is operator dependent and has a high incidence of artefacts in the evaluation of the upper ascending aorta. The interposition of the trachea and left main bronchus between the aorta and the oesophagus can cause a “blind spot” in this area, and also the presence of severe aortic wall calcifications may lead to misdiagnosis because of the resulting “acoustic shadows”. Additionally, the examination may be undetermined by the lack of a suitable acoustic window due to body habitus and artefacts related to the presence of prostheses. Furthermore, the use of TTE does not permit the visualization of the descending aorta. 

 The introduction of the latest generation of 64-slice CT scanners with short acquisition times and their high spatial and temporal resolution allows images to be acquired during a single short breath-hold and thus with a high level of anatomical detail of the whole thoracic aorta and of the other anatomic thoracic structures. Furthermore, ECG-gated synchronization with its reconstruction algorithms permits a correct evaluation of cardiovascular structures minimizing cardiac motion artefacts; the advantage of cardiac CT in these patients is that it allows a better visualization of the aortic root and aortic valve and that permits a simultaneous evaluation of the coronary arteries. A heart rate <70 bpm, whether spontaneous or induced by *β*-blockers, is considered sufficient to increase diastolic time and duration of end-diastole, when the heart, the valves, the aortic root, and coronary arteries are nearly motionless. The volume of data acquired during scanning time can be subsequently reelaborated by a postprocessing procedure. Multiplanar reconstruction (MPR), Maximum intensity projection (MIP), and volume rendering (VR) techniques provide a comprehensive depiction of the prosthesis valve, the aortic root, the thoracic aorta, and coronary artery tree. All this information is achieved with values of an absorbed dose of between 8–10 mSv. However, when the heart rate is stable and lower than 65 bpm, a prospective ECG-gated CT protocol could be used with a significant reduction in the adsorbed dose, up to 3–5 mSv, without decreasing the image quality. 

In our study we investigated the clinical applicability and image quality of retrospectively ECG-gated 64 slices CT for the followup of patients with aortic valve replacement due to BAV, in comparison with transthoracic echocardiography. In particular, our evaluation included thoracic aorta diameters at different levels: aortic root, sinotubular junction, ascending aorta, and aortic arch. In all patients the study was also extended to the descending aorta and completed with an evaluation of the coronary artery tree. We observed a statistically significant difference between CT and TTE in the evaluation of ascending aorta diameters. In particular, in 7 patients, the ascending aortic diameter measured by CT exceeded 50 mm, while echocardiographic measurements were lower. These results disagree with those of other studies, in particular the study by Tamborini et al. [19], in which there was a good correlation between CT and TTE measurements. 

Moreover, it is important to notice that in these patients we observed the longest followup overall, 60 months. This evidence underlines the progression of ascending aorta dilatation and also enhances the importance of long-term evaluation, with an accurate imaging technique, in patients with a prosthesis substitution of BAV.

The most important issue emerging from our study is that ascending aorta shows a progressively long-term dilatation while aortic root's diameters seem to be more stable. Probably, this happens because in patients with BAV syndrome the defects of the valve had previously caused a hemodynamic stress of the ascending aorta wall associated with a congenital alteration of the structures of the aortic wall.

## 5. Conclusion

Due to the different findings between CT and TTE studies, angio-CT should no longer be considered as a complementary exam in the followup of these patients, but as a fundamental role since it is a real necessity. We think that should be necessary to perform two angio-CT exams in followup, respectively 12 months and 48 months after aortic valve replacement. Indeed, if between these observation times there are changes in the clinical history of the patient, it is important to perform also one more angio-CT study. Our study demonstrated that angio-CT is a compulsory diagnostic step, in the followup of patients with previous BAV who have undergone aortic valve replacement, to avoid late complications. The information provided by angio-CT regarding the thoracic aorta is more complete and exhaustive than that obtained by TTE. 

## Figures and Tables

**Table 1:** Preoperative characteristics.

Variables	67 patients
Age (years)	61 ± 15
Male	48 (72%)
Body surface (m^2^)	1.86 ± 0.22
Body max index (kg/cm^2^)	27.2 ± 4.6
NYHA I-II class	38 (57%)
NYHA III class	21 (31%)
NYHA IV class	8 (12%)
Family history of cardiovascular disease	24 (36%)
Hypertension	39 (58%)
Smoking status	27 (40%)
Obesity	17 (25%)
Hypercholesterolemia	9 (13%)
Hypertriglyceridemia	10 (15%)
Diabetes mellitus	10 (15%)
Chronic renal failure	1 (15%)
Chronic obstructive pulmonary disease (COPD)	3 (4%)
Chronic obliterative arterial disease of lower limbs	13 (19%)
Recent myocardial infarction (<30 days)	2 (3%)
Previous myocardial infarction (>30 days)	5 (8%)
Previous transluminal coronary angioplasty	3 (4%)
Atrial fibrillation	1 (1.5%)
Obstructive coronary artery disease	10 (15%)
Single-vessel coronary artery disease	6 (9%)
Double-vessels coronary artery disease	4 (6%)
Pure aortic valve stenosis	7 (10%)
Mixed aortic valve stenoinsufficiency	23 (35%)

**Table 2:** Preoperative echocardiographic parameters.

Variables	67 patients
Left ventricle	
Telediastolic diameter (mm)	54.9 ± 9.6
Telesystolic diameter (mm)	37.3 ± 10.1
Interventricular septum (mm)	13.9 ± 2.8
Posterior wall (mm)	13.0 ± 2.4
Ejection fraction (%)	55.2 ± 12.2
Aorta	
Annulus (mm)	24.1 ± 5.4
Root (mm)	36.4 ± 4.1
Sino-tubular junction (mm)	33.0 ± 4.9
Ascending tract (mm)	37.5 ± 4.4
Maximum transvalvular gradient (mmHg)	85.6 ± 26.4
Transvalvular average gradient (mmHg)	54.1 ± 17.7
Aortic valve regurgitation (1→4)	1.1 ± 1.1
Mitral valve regurgitation (1→4)	0.4 ± 0.7
Tricuspid valve regurgitation (1→4)	0.1 ± 0.4
PAPs (mmHg)	33.8 ± 6.3

**Table 3:** Preoperative angio-CT ascending aorta parameters.

Variables	67 patients
Aortic root	38.22 ± 5.3
Ascending aorta	40.2 ± 3.9
Aortic arch	28.5 ± 7.7

**Table 4:** Clinical characteristics at followup.

Variables	67 patients
Age (years)	64.3 ± 14.4
NYHA I-II Class	56 (84%)
NYHA III-IV Class	11 (16%)
Mortality at followup	0
Aortic dissection	0
Aortic rupture	0
Thromboembolic or hemorrhagic episodes	8 (12%)
Infectious endocarditis	1 (1.5%)
Major cardiovascular events	7 (10%)
Paroxysmal Atrial fibrillation	2 (3%)
Malignant ventricular tachycardia	2 (3%)
Atrioventricular block grade III	1 (1.5%)
Atrial fibrillation at high frequency	2 (3%)

**Table 5:** Echocardiographic parameters at followup.

Variables	67 patients
Left ventricle	
Telediastolic diameter (mm)	50.8 ± 6.0
Telesystolic diameter (mm)	34.4 ± 6.7
Interventricular septum (mm)	12.9 ± 2.1
Posterior wall (mm)	12.1 ± 1.6
Ejection fraction (%)	54.8 ± 8.8
Aorta	
Annulus (mm)	24.5 ± 3.2
Root (mm)	36.7 ± 4.1
Sino-tubular junction (mm)	34.5 ± 3.1
Ascending tract (mm)	39.6 ± 4.8
Maximum transprosthetic gradient (mmHg)	24.4 ± 9.7
Transprosthetic average gradient (mmHg)	13.3 ± 5.4
Aortic valve regurgitation (1→4)	0.2 ± 0.5
Mitral valve regurgitation (1→4)	0.4 ± 0.6
Tricuspid valve regurgitation (1→4)	0.3 ± 0.7
PAPs (mmHg)	30.8 ± 5.6

**Table 6:** Angio-CT ascending aorta parameters at followup.

Variables	67 patients
Thoracic Aorta	
Root	37.9 ± 5.5
Ascending tract	43.1 ± 5.2
Arch	31.2 ± 5.7
Descending tract	28.9 ± 3.8
